# Mechanisms of Blood–Brain Barrier Protection by Microbiota-Derived Short-Chain Fatty Acids

**DOI:** 10.3390/cells12040657

**Published:** 2023-02-18

**Authors:** Ekaterina Fock, Rimma Parnova

**Affiliations:** Sechenov Institute of Evolutionary Physiology and Biochemistry, Russian Academy of Sciences, 194223 St. Petersburg, Russia

**Keywords:** short-chain fatty acids, blood–brain barrier, gut microbiota, tight junction proteins, histone deacetylase, receptors, nuclear factor kappa B, nuclear erythroid 2-related factor 2

## Abstract

Impairment of the blood–brain barrier (BBB) integrity is implicated in the numerous neurological disorders associated with neuroinflammation, neurodegeneration and aging. It is now evident that short-chain fatty acids (SCFAs), mainly acetate, butyrate and propionate, produced by anaerobic bacterial fermentation of the dietary fiber in the intestine, have a key role in the communication between the gastrointestinal tract and nervous system and are critically important for the preservation of the BBB integrity under different pathological conditions. The effect of SCFAs on the improvement of the compromised BBB is mainly based on the decrease in paracellular permeability via restoration of junctional complex proteins affecting their transcription, intercellular localization or proteolytic degradation. This review is focused on the revealed and putative underlying mechanisms of the direct and indirect effects of SCFAs on the improvement of the barrier function of brain endothelial cells. We consider G-protein-coupled receptor-mediated effects of SCFAs, SCFAs-stimulated acetylation of histone and non-histone proteins via inhibition of histone deacetylases, and crosstalk of these signaling pathways with transcriptional factors NF-κB and Nrf2 as mainstream mechanisms of SCFA’s effect on the preservation of the BBB integrity.

## 1. Introduction

The blood–brain barrier (BBB) isolates the brain parenchyma from the bloodstream and maintains the brain homeostasis. Due to its specific structural and functional organization, the BBB provides the highly active bidirectional metabolic exchange between the microvasculature and the brain, and simultaneously restricts paracellular and transcellular transport into the brain. Low BBB permeability is largely provided by endothelial cells, lining the cerebral microvasculature, in close functional interactions with neurons, astrocytes and pericytes which together form the so-called neurovascular unit (NVU) [1,2,3].

Dysregulation of the barrier permeability induced by multiple pathological stimuli can cause the infiltration of leukocytes, influx of water and plasma proteins, passage of bacteria and their toxic products, entry of pro-inflammatory mediators leading to activation of glial cells, inflammation and neuronal dysfunction. Numerous neurological disorders associated with aging, neuroinflammation and neurodegeneration, such as Alzheimer’s disease, Parkinson’s disease, multiple sclerosis, epilepsy, as well as behavioral and mental impairment are accompanied, or even primarily caused, by BBB leakage (for reviews, see [4,5,6]). Microbial infection, systemic inflammatory conditions, diabetes, obesity and cancer also led to progressive leakiness of the BBB worsening the course of the disease [6]. In addition, stress, unbalanced diet, antibiotics, high salt consumption and other factors can also negatively affect the BBB permeability [7,8,9].

It is now evident that among other factors, gut dysbiosis can cause the impairment of the BBB integrity and brain dysfunction. The so called “gut–brain axis”, consisting of a complex network of immune, neuronal, and endocrine signaling pathways, provides bidirectional communication between the nervous system and gastrointestinal tract. Gut microbiota dysbiosis associated with intestinal inflammation and increased intestinal permeability has been revealed in numerous neurological, mental and behavioral dysfunctions, such as cerebral ischemia, Alzheimer’s and Parkinson’s diseases, depression, autism spectrum disorder and others (for reviews, see [10,11,12]).

One of the most important microbial metabolites that link the alterations in gut microbial composition and brain dysfunction are short-chain fatty acids (SCFAs), mainly butyrate, propionate and acetate, produced by anaerobic bacterial fermentation of the dietary fiber in the intestine. In both animals and humans, SCFAs are known to be required for the normal functioning of numerous physiological processes, such as innate and adaptive immune reactions, metabolism, energy expenditure, intestinal and vascular integrity, circadian rhythms, sleep, appetite and others [10,13,14,15]. Their multiple cellular effects are mediated by binding to G-protein-coupled receptors (mainly GPR43, GPR41, GPR109A) and by inhibition of histone deacetylation, causing epigenetically regulated alterations in gene expression [16,17]. In the CNS, SCFAs play a critical role in neurodevelopment, prevention of neuroinflammation, maturation of microglia, neurogenesis in the adult brain, modulation of neurotransmitters and neurotrophic factor production, and memory consolidation [10,18,19,20,21].

Manipulating the gut bacterial microbiome, such as an application of antibiotics that specifically alters the gut bacteria composition, probiotic administration, fecal microbiota transplantation and replacing the lacking microbiome with SCFAs only (germ-free mice), revealed that SCFAs are essential for the maintenance of the BBB integrity in normal and pathological conditions [9,22,23,24,25,26], although other microbiota-derived metabolites, such as trimethylamine-*N*-oxide and amino acid derivatives, can also influence the integrity of the BBB [27,28]. A number of preclinical works indicate that oral administration of SCFAs promotes the recovery of the compromised BBB in various CNS pathologies, such as hypoxia–ischemia conditions [29,30], traumatic brain injury, sepsis-associated encephalopathy [31] and Parkinson’s disease [32]. Data on an in vitro BBB model showed that the protective effect of SCFAs on BBB integrity is provided by their direct action on endothelial cells and activation of anti-inflammatory and anti-oxidative cellular pathways [33].

Given the exclusive importance of the BBB for brain homeostasis, maintenance of the BBB functional and structural integrity by SCFAs can be considered as one of the most important mechanisms of their neuroprotective action. In this review, we have brought together the in vitro and in vivo data concerning the protective effect of SCFAs on BBB integrity. Firstly, we briefly considered the current knowledge of the mechanisms of intercellular junctional complex disturbances in response to inflammatory stimuli as a main mechanism for BBB leakiness. Then, we focused on revealed and putative mechanisms of SCFA action on brain endothelial cells and other NVU components, such as astrocytes and microglia, highlighting the receptor-mediated effects of SCFAs, SCFA-induced inhibition of histone deacetylases and their crosstalk with transcriptional factors NF-kB and Nrf2 as mainstream signaling pathways. Given the similarity of the principal mechanisms of cellular barrier regulation in different tissues, we also considered the data on the mechanisms of the protective effect of SCFAs found in the epithelium and peripheral endothelium. Finally, we discussed the indirect effect of SCFAs on BBB integrity through suppression of peripheral inflammation and have presented schematic diagrams illustrating both revealed and potential mechanisms of SCFA protective action on brain endothelial cells.

## 2. Regulation of the BBB Paracellular Permeability in Normal and Pathological Conditions

**Figure 1:**
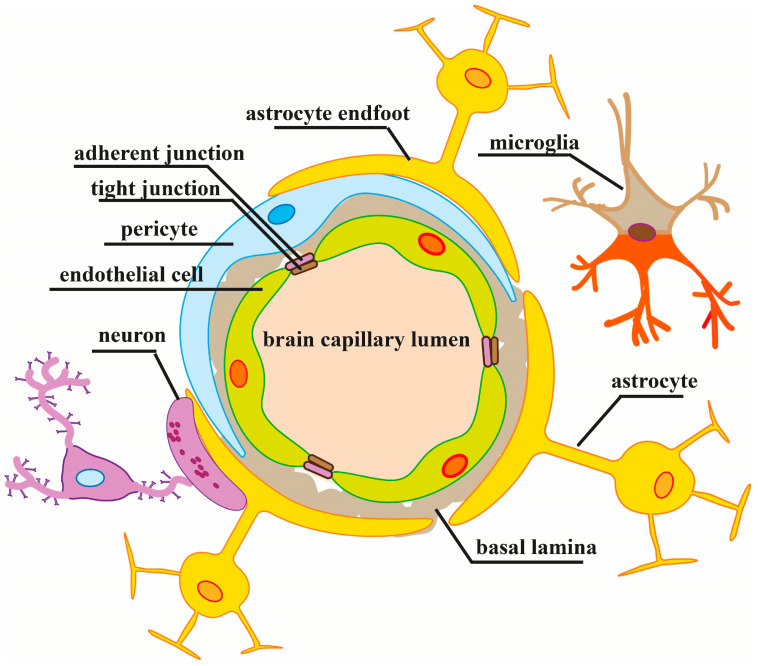
Organization of the neurovascular unit. The NVU is composed of endothelial cells, astrocytes and pericytes. The cerebral endothelial cells form the BBB providing low paracellular permeability via a set of tight and adherent junctions which connect adjacent endothelial cells. Pericytes partially surround the endothelium and, along with the endothelial cells, are enclosed in the basal lamina, a structure that provides mechanical support and functions as a barrier as well. Astrocyte endfeet are in close contact with endothelial cells and pericytes. The perivascular network of astrocytes connects the blood vessel with neurons. The NVU components in cooperation with perivascular microglia intimately interact with each other via anatomical relationships and reciprocal chemical signaling mediating brain function in normal and pathological conditions.

The cerebral endothelial cells play a major role in maintaining the BBB integrity via junctional complexes between the adjacent cells creating a high-resistance paracellular barrier to molecules and ions. However, other NVU members, pericytes, astrocytes and neurons, in cooperation with perivascular microglia and the extracellular matrix, are also critically important in the regulation of BBB permeability and brain homeostasis through multidimensional, continuous and reciprocal interconnections among all NVU components via physical contacts and released signaling mediators (for reviews, see [2,34]). A NVU organization scheme is shown in Figure 1.

The barrier properties of the cerebral endothelium differ from those of the periphery due to the particular features of its structural and molecular organization, such as specific intercellular junction complexes which limit passive paracellular transport, lack of fenestration and low rates of vesicular transport, highly selective influx and efflux transport, high numbers of mitochondria, a thick luminal glycocalyx layer, a low level of expression of leukocyte adhesion molecules, enrichment of the plasma membrane with long-chain polyunsaturated fatty acids and other functions [6,35,36,37,38,39].

The mechanisms of the BBB disruption can include loss of tight junction integrity, increase in transcytosis, endothelial cell apoptosis, breakdown of glia limitans, degradation of the glycocalyx and other mechanisms [6]. Here, we briefly overview only the regulation of paracellular permeability of the BBB which is restricted by intercellular junctional complexes between endothelial cells. These complexes are composed of a branching network of sealing strands consisting of tight junctions (TJs), adherens junctions (AJs) and gap junctions (GJs) which are intimately linked and continuously crosstalk. TJ proteins (TJPs), notably claudins, occludin and junction adhesion molecule (JAM), form homophilic interactions with corresponding proteins on adjacent endothelial cells sealing up the paracellular space. Cytoplasmic scaffold proteins, zonula occludens-1, -2, -3 (ZO-1/2/3), and some other proteins link intracellular domains of TJ proteins with the actin cytoskeleton, which implements the stabilization of TJ strands [36,40,41,42].

The components of AJ complexes, e.g., vascular endothelial (VE)-cadherin, platelet endothelial cell adhesion molecule-1 (PECAM-1) and nectin, carry out cell–cell adhesion by their homophilic interactions between adjacent cells. AJ proteins are associated with the actin cytoskeleton via adaptive molecules, which maintain the structural and functional integrity of the endothelium. GJs are formed by connexins, the hemichannels located in the endothelial membranes, which allow small signaling molecules and ions (e.g., Ca^2+^) to move and transduce signals through the intercellular space [36,41].

The structural and functional integrity of the junctional complexes is dynamically regulated by various mechanisms, such as (1) caveolae-mediated internalization of TJP complex with subsequent recycling or degradation in lysosomes or proteasomes, (2) posttranslational protein modifications, (3) cleavage of TJ proteins by matrix metalloproteinases (MMPs) and (4) transcription/translation modulation [37,43,44]. All of these mechanisms can be involved in the destruction of junctional complexes and BBB “opening” under pathological stimuli.

Multiple signaling pathways activated by physical stimuli, such as shear stress, and by chemical factors circulating in the blood or produced by BBB-associated cells, regulate the molecular structure of junctional complexes. One of the most extensively studied is post-translational protein modification, such as phosphorylation, polyubiquitination, palmitoylation, glycosylation, acetylation and methylation, which regulate the functioning of TJ proteins, as well as scaffold and cytoskeleton proteins, affecting junctional complex assembly/ disassembly, protein trafficking and localization [38,44,45]. Various kinases (PKA, different PKC isoforms, PKG, PI3K, p38MAPK, JNK, RhoK, (PI3K)/Akt, AMPK, receptor and non-receptor PTK) and phosphatases were shown to be involved in the assembling/disassembling of TJ complexes [5,46].

In addition to transcription factors, epigenetic mechanisms, such as DNA methylation and histone acetylation, modify junctional complex gene expression [47,48]. Additionally, multiple steps of the synthesis and assembly of junctional complex proteins can be post-transcriptionally regulated by various miRNAs [48,49].

The reduction in TJ protein abundance is one of the most important mechanisms of BBB “opening” under pathological conditions. Various endogenous factors, such as IL-1β, IL-6, TNF-alpha, oncostatin M, prostaglandin E2, thromboxane A2, lysophospholipids, microRNAs and serum amyloid A, impair the BBB through this mechanism [5]. Restoration of BBB integrity induced by different chemical factors, including SCFAs, was shown to require the recovery of TJ protein expression.

## 3. SCFAs and Their Impact on Brain Health

SCFAs are saturated fatty acids with an aliphatic tail of 1–6 carbon atoms in a straight or branched-chain configuration (acetate, propionate, butyrate, isobutyrate, valerate, isovalerate and caproate). The main source of SCFA entry into the mammalian organism is the fermentation of dietary indigestible and non-absorbable fibers by the colonic bacteria. SCFAs are produced by various bacterial genera that are commonly found in the intestinal flora, such as Bifibacterium, Lactobacillus, Bacteroides, Ruminococcus, Firmicutes and others [14,15]. Minor amounts of SCFA originate from the food (for example, cow’s milk) and from the host metabolism. Acetate, propionate, and butyrate make up over 95% of the total SCFAs produced by the microbiota. These fatty acids are present in millimolar concentrations in the colon, and their molar ratio is approximately 4:1:1 [50]. Following their formation, SCFAs are absorbed by colonocytes, where they can be metabolized and extensively used as a fuel. Non-metabolized SCFAs enter the portal circulation. In the liver, SCFAs are further metabolized, and only a small amount enters the systemic circulation and reaches peripheral tissues. The SCFA circulating levels and their availability are highly dependent on dietary fiber intake and on the number of SCFA-producing bacteria in the colon [51,52]. Human plasma concentrations of acetate, propionate and butyrate are in the micromolar range: 19–150 µM of acetate, 1–13 µM of propionate or butyrate [53,54,55]. In the mouse plasma, butyrate and propionate concentrations are similar to those of humans (4–20 µM and 2–6 µM, respectively), whereas the acetate level is significantly lower (3–25 µM) [56].

SCFAs enter endothelial cells via monocarboxylate transporters (MCTs). In human and rodent brain endothelium, MCT1 (also known as SLC16A1), which acts as a proton- dependent cotransporter/exchanger, plays a key role in the entry of SCFAs into the brain parenchyma [57,58]. As has been shown in rat brain tissue sections, MCT1 was nearly equally distributed on both the luminal and abluminal endothelial cell plasma membranes [59,60].

Acetate, propionate and butyrate are also the substrates for electrogenic, sodium-dependent monocarboxylate transporter 1 (SMCT1, also known as SLC5A8), which is highly expressed in neurons but not in the BBB cells [14]. In an in vitro BBB model, it was shown that butyrate transfer is also mediated by FAT/CD36 (fatty acid translocase/CD36), a transporter specific for the transfer of medium- and long-chain fatty acids. Silencing of FAT/CD36 led to a 50% decrease in butyrate movement across the endothelium monolayer [61]. Passive paracellular diffusion of SCFAs is prevented by tight junction complexes, and in the healthy barrier, this seems to be of minor importance.

The brain takes up plasma SCFAs very rapidly. Experiments with ^14^C-butyrate injected into the carotid artery of rats showed that already 15 s after the label injection, the concentration of butyrate in the brain reached 46% of the plasma level [62]. The comparison of 10 min butyrate uptake by various organs showed that liver and spleen absorb butyrate most intensively. Its uptake by the brain was 6 times lower but was at the same level as in the heart [62].

An average content of 17.0 pmol/mg of tissue for butyrate and 18.8 pmol/mg of tissue for propionate in the human brain was reported [63]. Afterwards, these data were interpreted in the literature [14,20] as very low levels of SCFAs in the brain compared to their concentration in the plasma. However, this does not seem to be true. Recalculation of the data from [63] to brain water, which is approximately 75% of brain weight, results in higher levels of both SCFAs, i.e., 21 µM for butyrate and 23 µM for propionate, which are of the same order of magnitude as in plasma.

Decreased plasma or fecal levels of SCFAs, mainly butyrate, may be considered as a biomarker of different neurological disorders. The complex neuroendocrine and neuroimmune mechanisms closely interconnected with the functioning of the autonomic and enteric nervous systems provide a causal relationship between gut microbiota and brain function [20,64]. At least three types of SCFA receptors are expressed in intestinal epithelium (see below), which regulate the secretion of gut hormones and neurotransmitters, thereby communicating with the brain through the circulation or vagus nerve activation. A reduction in SCFA-producing colon bacteria, and subsequently SCFA content, has been observed in many neuropathological states such as multiple sclerosis [65], stroke [66], traumatic brain injury [52], vascular dementia [24], sepsis-associated encephalopathy [67], Alzheimer’s [68] and Parkinson’s diseases [69]. In most cases, administration of butyrate-producing bacteria or butyrate alone increased a positive neurological outcome [22,29,30,32,70,71]. Notably, a decrease in the number of SCFA-producing bacteria can be a cause, as well as a result of brain pathology, as happens for example after traumatic brain injury, highlighting bidirectional communication between gut and brain.

SCFAs are critically important in the maintenance of the health status of the intestine. Impaired gut immunity and loss of the intestinal epithelium integrity due to SCFA deficiency [72,73] allow bacteria, LPS and other toxic products to enter the circulation and initiate systemic inflammation leading to the impairment of various organs, including brain. Butyrate, in addition to being a major energy substrate for colonocytes, stimulates the secretion of mucin, which prevents LPS absorption, suppresses inflammation of epithelial cells, increases the activity of antioxidant systems and improves barrier integrity via an increase in the expression of TJ proteins [72]. As we discuss below, the protective effect of butyrate and other SCFAs on the integrity of BBB is provided by similar mechanisms.

## 4. Effect of SCFA-Produced Microbiota on BBB Integrity

The beneficial effect of SCFA-producing gut microbiota on the integrity of the BBB has been demonstrated in germ-free mouse models and with antibiotics that specifically alter the abundance of certain bacterial families. Thus, decreased expression of occludin and ZO-1 mRNAs in brain microvessels and increased BBB permeability were observed in mice treated with a mixture of 5 non-absorbable antibiotics which decrease the relative content of SCFA-producing genera in the gut. Fecal microbiota transplantation in antibiotic-treated mice with pathogen-free gut microbiota restored TJ protein expression and BBB integrity [23]. In agreement with this study, claudin-5 and occludin mRNA expression was reduced in the amygdala and hippocampus of mice treated with an antibiotic mixture. These changes were accompanied by reduction in acetate, propionate and butyrate content in the colon and by impairment of object recognition memory [26]. Oral but not intravenous administration of amoxicillin antibiotic, by altering gut microbiota composition, caused a significant increase in BBB permeability in rhesus monkeys [9]. Similar results with the use of an antibiotic mixture were obtained in mice subjected to anesthesia/surgery. Administration of a Lactobacillus bacterial mixture restored the level of claudin-5, occludin and ZO-1 expression in the hippocampus and improved the BBB permeability [71].

On the other hand, some antibiotics demonstrated an opposite effect. For example, in mice, the antibiotic cefazolin, by altering gut bacteria composition, elevates plasma acetate and propionate levels and improves the BBB barrier function damaged by anesthesia and surgery [56]. Low-dose penicillin used in late pregnancy and in early postnatal life induced long-term effects in the offspring of mice [74]. They exhibited impaired social behavior and displayed aggression, which were accompanied by an increase in the level of pro-inflammatory cytokines in the frontal cortex. These behavior alterations were prevented by supplementation with Lactobacillus rhamnosus, an SCFA producer. Surprisingly, that early-life penicillin treatment led to increased mRNA/protein expression of occludin and claudin-5 in the hippocampus. The authors speculated that the reinforcement of BBB integrity could prevent the production of inflammatory cytokines in the hippocampus, and this may be a protective mechanism to preserve hippocampus-dependent tasks, such as learning and memory [74].

In a mouse model of traumatic brain injury, it has been shown that intragastrically administered Clostridium butyricum, one of the most efficient butyrate producers, improved neurological dysfunction, brain edema, neurodegeneration and BBB impairment. A significant increase in the expression of occludin and ZO-1 was found in the brain of bacteria-treated mice [22]. Using germ-free mice, Braniste and coauthors showed that the BBB of these animals is characterized by a high permeability associated with the decreased expression of occludin and claudin-5 that was intensified from birth to adulthood [25]. Transplantation of pathogen-free gut microbiota, administration of Clostridium butyricum or application of sodium butyrate (SB) alone augmented BBB permeability to the level of that of the pathogen-free adult mice and reversed the decrease in the expression of occludin and claudin-5 in the frontal cortex and hippocampus [25]. These experiments indicate that microbiota-produced SCFAs play a key role in restoration and maintenance of BBB integrity.

## 5. Protective Effect of SCFAs on BBB Integrity in In Vitro BBB Models and in In Vivo Animal Models of CNS Pathologies

Below, we summarize publications that demonstrated the effects of SCFAs on BBB integrity in in vitro BBB models and in vivo in various neurodegenerative, neuroinflammatory and acute brain injury animal models (Table 1). In all the cited papers, widely accepted approaches to assessing BBB permeability, such as the use of paracellular tracer compounds and characterization of TJ mRNA/protein expression, were applied. In an in vitro BBB model, transendothelial electrical resistance (TEER), which reflects the ionic conductance of the endothelium layer, was additionally assessed.

Back in the late 1990s, Hurst and Clark created an in vitro BBB model by coculturing non-brain vascular endothelial cells with glioma cells, which leads to differentiated endothelial cell phenotype with BBB properties [75]. They showed that butyrate at doses close to physiological (20–80 μM) significantly enhanced transendothelial electrical resistance (TEER), wherein this effect only occurred in endothelial/glioma cell co-culture and an exposure of more than 6 h, suggesting specific protein synthesis. Much later, using hCMEC/D3 as a human BBB in vitro model, Hoyles and coauthors found that propionate and butyrate but not acetate significantly attenuated the LPS-induced decrease in TEER and increase in paracellular tracer permeability. Propionate alleviated the LPS-induced marked disruption in intracellular localization of occludin, claudin-5 and ZO-1 [33]. In the mouse in vitro BBB model (bEnd.3 cells), butyrate and propionate used at physiologically relevant concentrations (1 μM) restored the LPS-induced decrease in ZO-1 and claudin-5 localization at the cell–cell junctions and increased the area occupied by mitochondria, suggesting prevention of LPS-induced impairment of brain endothelial cell mitochondrial dynamics [76]. Interestingly, although both fatty acids increased TEER, no changes in mRNA levels of occludin, claudin-5 and ZO-1 were observed [76].

SCFAs administration mitigated the course of severe pathological states such as stroke, sepsis and traumatic brain injury. Stroke leads to significant damage of the BBB integrity disrupting TJs, activating inflammation and infiltration of peripheral immune cells into parenchyma [79]. In rats subjected to MCAO induced by intracerebral injection of endothelin-1, SB administered 6 h after stroke increased positive neurological outcomes and attenuated BBB disruption, as assessed by increased serum level of glial fibrillary acidic protein, a biomarker of brain injuries [29]. Wang and coauthors showed that postischemic SB treatment remarkably attenuated MCAO-induced BBB disruption, while reducing elevation of MMP-9, degradation of TJ proteins and nuclear translocation of NF-κB [77]. Traumatic brain injury-induced neurological deficits, brain edema and neuronal damage, increase in BBB permeability and the decrease in occludin and ZO-1 protein expression were all attenuated by SB administration [30].

The neuroprotective effect of an SCFA mixture has been recently demonstrated in a mouse model of sepsis induced by cecal ligation and puncture [31]. Importantly, the level of acetate, propionate and SCFA-producing bacteria in the fecal samples from the septic mice were significantly lower than those of the control group. Besides attenuation of the behavioral impairment and neuronal degeneration, SCFA mixture protected the BBB integrity by upregulating the expressions of occludin and ZO-1. In a mouse model of Parkinson’s disease, SB was shown to attenuate disease-associated disruption of the BBB by upregulation of occludin and ZO-1. These effects were accompanied by improved neurobehavioral impairment including cognitive abilities, coordination performance and dopaminergic neuron degeneration [32].

SB improved postoperative cognitive function in aged mice with gut dysbiosis. This positive outcome was associated with SB-induced reduction in BBB permeability and an increase in occludin, claudin-5 and ZO-1 protein expression in the hippocampal tissue [71]. Feeding mice with a high fructose diet (30% fructose for eight weeks) combined with chronic stress was found to cause neuroinflammation, hippocampal neurogenesis decline and BBB damage promoting the development of depressive-like behavior. The brain vasculature of these mice displayed a significant decrease in occludin, claudin-5 and ZO-1 protein expression. Oral administration of a SCFA mixture rescued the BBB decline by significantly increasing the TJ proteins expression [7]. Dietary supplementation with monobutyrin glycerol ester to the diet of high-fat-fed and ApoE-deficient rats was found to strengthen the BBB by up-regulating occludin and ZO-1 expression in the brain [78].

The in vivo data mentioned above indicate that the SCFA-induced decrease in brain endothelial permeability and recovery of TJ protein expression are an important part of the overall positive neurological outcome, with a causal relationship specific to each pathological condition. However, data obtained on the hCMEC/D3 cell line, definitively provide evidence that SCFAs are capable of protecting BBB integrity via their direct interaction with brain endothelial cells. The described results show that the protective effect of SCFAs on the BBB integrity is related to the restoration of TJ protein function/expression, leading to a decrease in permeability. Such a mechanism of the SCFA effect is likely to be common for various types of tissue barriers, since it has been demonstrated in the intestinal [80,81,82] and ruminal epithelia [83,84] and in peripheral endothelium [85,86]. Among microbiota-derived SCFAs, butyrate seems to be the most important in the preservation of the barrier integrity.

## 6. SCFAs as Ligands of G Protein-Coupled Receptors

SCFAs are endogenous ligands for G protein-coupled receptors, GPR41 (or FFAR3), GPR43 (or FFA2) and GPR109A (or hydroxycarboxylic acid receptor 2, HCAR2), which belong to the family of nutrient-sensing receptors. GPR41 and GPR43 share 52% similarity and are activated by acetate, propionate, butyrate, and pentanoate with different specificity for carbon chain length. For GPR41 activation, propionate was the most potent, whereas for GPR43, propionate and acetate were equally potent and more effective than butyrate [16,87]. GPR41 is expressed ubiquitously in a wide diversity of tissues, including brain. In the human, the highest expression level was found in adipose tissue, where GPR41 was identified mainly in the endothelial cells of arteries and arterioles [16]. The expression of GPR41 has been confirmed immunochemically in endothelial cells of human brain capillaries, in the human cerebromicrovascular endothelial cell line hCMEC/D3 [33] and in the peripheral vasculature [17,88]. GPR41 was found in rat cortex neurons, and its expression was increased under butyrate administration [89].

Initially, GPR43 was found in immune cells, especially neutrophils and monocytes [16,87], and later, other sites of expression were found, e.g., intestinal epithelium, peripheral vasculature, brain, heart, adipose tissue, kidney, liver and others [17,88,90,91,92].

GPR41 and GPR43 exhibited a differential coupling to the downstream G proteins. GPR41 exclusively couples to Gαi/o, whereas GPR43 displays a coupling to Gαi/o and Gαq Gi/o [87]. Generally, coupling of both receptors induces inositol 1,4,5-trisphosphate formation, an increase in [Ca^2+^]_i_, ERK1/2 activation, and a decrease in cAMP [87]. In microglia and macrophages, GPR43 was shown to interact with β-arrestin-2, a scaffold protein for a great diversity of G-protein-coupled receptors, which regulates receptor desensitization and internalization [67,93].

Through GPR43 and GPR41 SCFAs exert various physiological effects in different cells regulating immunity, vascular tone, insulin and leptin production, intestinal homeostasis, gut hormone synthesis, sympathetic regulation of energy output and others (for a review, see [94]). Both GPR43 and GPR41 are expressed in different epithelial cells, such as intestinal [72,95], renal [96], ruminal [83] and lung alveolar [97], where they mediate the anti-inflammatory effect of SCFAs. GPR41 seems to contribute to the preservation of the barrier’s structural integrity. Using GPR41 knockdown bovine ruminal epithelial cells, it was found that GPR41 mediates the protective effect of SB against the LPS-induced increase in epithelial permeability and suppression of occludin, claudin-5 and ZO-1 expression [83]. In the same cellular model, a SCFA mixture significantly up-regulated the expression of GPR41 and many other genes involved in TJ structures. GPR41 knockdown cells exhibit an impairment of the bovine rumen epithelial barrier [84].

In the brain, GPR43 mediates the anti-inflammatory effect of SCFAs, as was shown in mouse neuropathological models, such as sepsis-associated encephalopathy [67], perioperative neurocognitive disorder [98] and Alzheimer’s disease [99].

GPR109A was initially identified as a receptor of the antidyslipidemic drug niacin (nicotinic acid) [100]. Later the ketone body 3-hydroxy-butyrate was identified as an endogenous ligand for GPR109A [101]. Butyrate is a low affinity GPR109A agonist (EC_50_ value of 0.7 mM), whereas propionate and acetate do not activate the receptor [101]. GPR109A is expressed in various barrier tissues, such as intestinal and mammary epithelium [80,102,103], in immune cells, adipocytes, brain (for a review, see [14]). Activation of GPR109A is coupled to the inhibitory G protein G_i_/G_o_ or to β-arrestins which can be recruited from the cytosolic compartment to the cell membrane [104].

In intestinal epithelium, GPR109A expression is stimulated by gut microbiota, since in germ-free mice, its expression was markedly reduced [105]. GPR109A(−/−) mice are highly susceptible to development of colonic inflammation and colon cancer [102]. Intestinal GPR109A can effectively bind butyrate due to its high concentration in the colonic lumen (in the millimolar range) and GPR109A-mediated downstream signaling is considered as one of the main mechanisms of the protective effect of SCFAs in the intestine [72]. Through binding to GPR109A, butyrate alleviates oxidative stress and inflammation in intestinal, and also in mammary gland epithelia, and controls barrier properties by enhancing the expression of TJ proteins [80,102,106].

The potential role of GPR109A in maintaining BBB integrity was established in a rat model of ketamine-induced psychosis, which is accompanied by BBB dysfunction. It was shown that activation of GPR109A by niacin, a major ligand for GPR109A, leads to the improvement of the inflammation and to the augmentation of the hippocampal expression of ZO-1, occludin and claudin-5 proteins [107]. These data indicate that GPR109A contributes to the control of the BBB permeability by an unknown mechanism. Expression of GPR109A in brain endothelial cells is still not confirmed. Additionally, it remains unclear whether endogenous butyrate, a low affinity GPR109A agonist, can activate the receptor in brain endothelial or other CNS cells, given that the plasma concentration of butyrate is less than 20 μM. This mechanism seems to be rather important in barrier tissues naturally exposed to SCFAs at a level significantly higher than in plasma, such as the intestine and mammary gland. Future investigations will clarify whether butyrate can activate GPR109A in the BBB.

## 7. SCFAs as Histone Deacetylase Inhibitors

Histone acetylation/deacetylation reactions are catalyzed by two counteracting protein families, histone deacetylases (HDACs) and histone acetyltransferases (HAT). The HDAC family consists of 18 different subtypes in four different classes: I (HDAC1-3 and 8), II (HDAC4-7 and HDAC9-10), III sirtuins (SIRT1-7) and IV (HDAC11) [108,109]. Removal of acetyl groups from lysine residues by HDACs increases chromatin compaction leading to transcriptionally silenced chromatin, whereas HDAC inhibition enhances histone acetylation, which results in chromatin relaxation facilitating transcription factor interaction with specific gene promoters. Besides histones, HDAC inhibition also promotes the acetylation of many other proteins [110].

A wide range of brain disorders is associated with imbalances in histone acetylation and transcriptional dysfunctions. It was shown in animal in vivo and in vitro models of acute injury and neurodegeneration that brain HDAC activity or expression is increased under neuropathological conditions, and various HDAC inhibitors demonstrate their neuroprotective, neurotrophic and anti-inflammatory properties (for reviews, see [109,111,112]). At the cellular and molecular level, the protective effect of HDAC inhibitors is associated with the prevention of excitotoxicity, inflammation, oxidative stress, mitochondria damage, ER stress, activation of MMPs and disruption of junctional complexes (for a review, see [109]).

Increased HDAC expression and histone hypoacetylation leading to BBB injury could play a pivotal role in ongoing post-injury BBB changes and recovery [48]. It was shown that HDAC3 or HDAC9 inhibition by specific inhibitors or via gene silencing protected the BBB from the injury attenuating the increase in permeability and down-regulation of TJ proteins induced by different pathological stimuli [48,77,113,114,115].

SCFAs are known to be inhibitors of HDAC activity and expression in various cell types. In in vitro experiments with partially purified histone-deacetylating enzymes, it was shown that butyrate, among other SCFAs, was the most potent inhibitor of histone deacetylation, while propionate and pentanoate were also effective [116]. In relation to butyrate, it was shown later that it is a competitive inhibitor of HDAC acting on a substrate binding site with a Ki of 46 µM. Butyrate blocks mainly HDAC class I, IIa and IV [108]. Data on the inhibitory potency of acetate in relation to HDACs are contradictory [116,117,118]. Additionally, SCFAs are able to lower HDAC expression [119].

A well-known HDAC inhibitor is a medium-chain fatty acid valproate (2-propylpentanoic acid), a traditional antiepileptic multitarget drug. Valproate increases histone acetylation and restores BBB TJ complexes in various pathological states, such as stroke [77], traumatic brain injury [120,121] and intracerebral hemorrhage [122].

The direct interaction between SCFAs and HDACs assumes that fatty acids should enter the cell. However, SCFA-induced HDAC inhibition may also be mediated through activation of surface receptors GPR43/GPR41/GPR109A and subsequent downstream pathways. Thus, in colonic regulatory T cells, propionate treatment enhanced histone acetylation, and this effect was dependent on GPR43 expression [123]. GPR41 mediates the effect of propionate on inhibition of HDAC activity/expression in hepatocellular carcinoma cells [124]. Butyrate-stimulated histone acetylation in mammary epithelial cells strongly depends on activation of GPR109A [106]. Additionally, both extra- and intracellular actions of SCFAs could take place in the same cell. For instance, in HUVEC culture exposed to inflammatory stimuli, butyrate and propionate suppressed IL-6 production via activation of GPR41/43, whereas these fatty acids decreased IL-8 and VCAM-1 expression through receptor-independent inhibition of HDAC [17].

Most authors who have studied the protective effect of SCFAs on BBB integrity consider that HDAC inhibition and subsequent alterations of gene transcription can play a central role in the recovery of TJ proteins and restoration of low BBB permeability [29,30,48].

## 8. Revealed and Putative Mechanisms Underlying SCFAs Protective Effect on the BBB

BBB injury associated with neurodegenerative and neuroinflammatory disorders is driven mainly by oxidative stress and inflammation induced by numerous inflammatory mediators that act both from the capillary lumen and the brain parenchyma. As in other barrier tissues, the mechanisms of the protective effect of SCFAs on BBB integrity are generally based on their antioxidant and anti-inflammatory actions mediated by inhibition of NF-κB and activation of Nrf2, a redox-sensitive transcription factor, important in counteracting the NF-κB-driven inflammatory response. Both nuclear factors compete for the same binding site in the nucleus and reciprocally influence each other’s expression and activity through a variety of mechanisms (for reviews, see [125,126]).

### 8.1. SCFAs/HDAC/NF-κB

**Figure 2:**
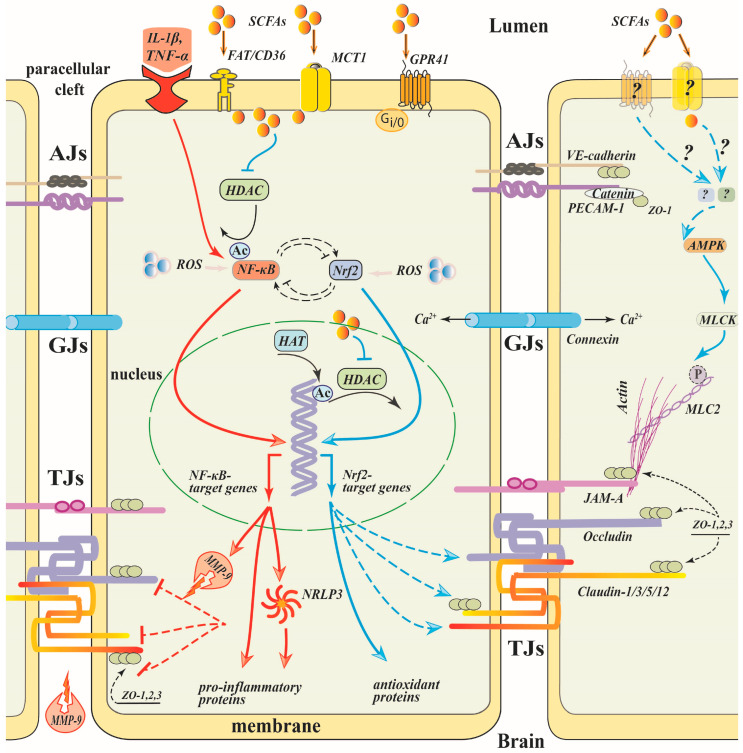
Mechanisms of SCFA effects on a brain endothelial cell (Part 1). Pathological stimuli activate NF-κB-mediated expression of multiple genes, including MMP-9 and NLRP3, and cause the degradation of TJ proteins. Activation of Nrf2 counteracts the NF-κB-driven inflammatory response. SCFAs suppress NF-κB and promote Nrf2 activation, leading to the recovery of TJPs. SCFAs can act after entry via transporters (MTC1, FAT/CD36) or via binding to receptors (e.g., GPR41). SCFAs inhibit HDAC, which results in an increase in histone acetylation, facilitating expression of genes, including Nrf2, that contribute to BBB integrity, and increase NF-κB acetylation, inhibiting its transcriptional activity. Under pathological stimuli, MLCK phosphorylates MLC2 to form actin stress fibers resulting in the endocytosis of transmembrane TJPs and loss of BBB integrity. SCFAs may promote the reassembly of TJs via suppression of the MLCK/MLC2 pathway. Dotted lines—putative signaling pathways. Blue lines—positive effects; red lines—negative effects. Symbols on the scheme—see in the list of abbreviations.

Numerous data provide evidence that inhibition of NF-κB transcriptional activity plays a central role in the anti-inflammatory effects of SCFAs, mainly butyrate, in various cellular types [67,93,127,128,129] (Figure 2). Excessive ROS production is a common trigger for the downstream pathways that mediate BBB leakage, rearrangement of the cytoskeleton and suppression of TJ proteins expression [130,131,132]. Oxidative stress triggers nuclear translocation of NF-κB/p65, that promotes the transcription of a great diversity of pro-inflammatory genes, such as cytokines, chemokines, adhesion molecules, COX2 and iNOS enzymes, which affect all NVU components and disrupt the BBB integrity by numerous mechanisms, including suppression of the assembly and expression of TJ proteins [31,133,134]. Inhibition of NF-κB activity restores the low permeability and upregulates the expression of TJPs, as was shown in different barrier tissues including the BBB [83,135,136,137]. Overexpression of the NF-κB/p65 alone repressed claudin 5 promoter activity in mouse brain endothelial cells [133]. SB was shown to inhibit NF-κB/p65 nuclear translocation alleviating inflammatory stimuli-induced damage of the barrier integrity in intestinal [137,138,139], bovine ruminal [79] and mammary [140] epithelia. Inhibition of NF-κB activation under propionate was observed in the brain microvascular cell line hCMEC/D3 [33].

The underlying mechanisms of the SCFAs effect on inhibition of NF-κB are poorly understood. NF-κB activity is known to be modulated by its post-translational acetylation that depends on the balance between the HDACs and the histone acetyltransferase activities [108,141]. Inhibition of HDACs can cause hyperacetylation of NF-κB/p65 leading to modulation of p65 binding to IκB and downregulation of NF-κB transcriptional activity [142]. Such a mechanism of NF-κB signaling interruption and subsequent protection of the BBB has been demonstrated in a rat model of cerebral ischemia upon administration of valproate [77], a broad HDAC inhibitor, or RGFP966, an HDAC3-specific inhibitor [143], suggesting that SCFAs, being HDAC inhibitors, can suppress NF-κB by the same mechanism (Figure 2). In the human colon adenocarcinoma cell line, Colo320DM, butyrate, propionate and acetate dose-dependently inhibited NF-κB reporter activity with the same rank of potency as HDAC inhibition (butyrate > propionate > acetate) [129].

### 8.2. SCFAs/GPR43/β-Arrestin-2/NF-κB

The GPR43 receptor engages a signaling pathway mediated by β-arrestin-2 which directly interacts with IκB, the NF-κB inhibitor, and blocks IκB phosphorylation/degradation, thereby suppressing NF-κB downstream signaling [144,145]. GPR43/β-arrestin-2-mediated suppression of NF-κB signaling pathway has been shown to be a mechanism of the anti-inflammatory effect of SCFAs in macrophages [93] and microglia [67]. As far as we know, the expression of GPR43 in brain microvessels has not yet been confirmed, so this mechanism may be rather important for the indirect effect of SCFAs on the BBB through the activation of GPR43 in microglia (see below).

### 8.3. SCFAs/NF-κB/NLRP3 Inflammasome

Activation of NF-κB induces NOD-, LRR- and the pyrin domain-containing protein 3 (NLRP3) inflammasome that causes the amplification of the inflammatory response, mainly by induction of IL-1β and caspase-1 expression [146]. The NLRP3 inflammasome has been identified as a mediator of BBB disruption in sepsis-associated encephalopathy [147]. In brain microvessel epithelial cells treated with LPS, downregulation of NF-κB-mediated NLRP3 activation was shown to restore TJ protein expression and cell monolayer permeability [147]. SCFA-induced suppression of the NLRP3 inflammasome, resulting in the restoration of the barrier function and TJ protein expression, has been shown in the intestinal epithelium [139,148,149].

### 8.4. SCFAs/NF-κB/MMP-9

NF-κB activation leads to upregulation of MMP-9, a member of the zinc-dependent endopeptidase family [77,135,150]. In brain tissues, MMP-9 is a critically important contributor to BBB damage (Figure 2). Under the action of pro-inflammatory cytokines, MMP-9 can be activated and secreted by recruited neutrophils [151], pericytes, microglia, and brain microvascular endothelial cells resulting in the proteolytic damage of the extracellular matrix components, degradation of the basement membrane and TJ proteins [135,136]. Both in vivo and in vitro data indicate that down-regulation of MMP-9 expression or activity in endothelial cells restores BBB disruption and elevates TJ protein expression [135,136,152,153]. In brain microvascular endothelial cells, IL-1β-induced MMP-9 expression occurs via complex signaling pathways including ROS-triggered c-Src-mediated transactivation of the EGF receptor and subsequent upregulation of MAP-kinases (e.g., ERK1/2, p38, and JNK1/2), resulting in turn in activation of NF-κB and MMP-9 expression [154].

Downregulation of the NF-κB/MMP-9 signaling pathway by SCFAs was demonstrated in a rat focal cerebral ischemic model [77]. I.p.-injected butyrate significantly reduced nuclear translocation of NF-κB, strongly inhibited MMP-9 protein expression and activity followed by the restoration of protein levels of claudin-5 and ZO-1 in cortex and striatum [77]. The inhibition of the NF-κB/MMP-9 pathway by butyrate was also observed in other cell types, such as IL-1β-inflamed chondrocytes [155].

### 8.5. SCFA/Keap-1/Nrf2 Signaling Pathway

SCFA-induced suppression of excessive ROS production and oxidative stress observed in endothelial and other cell types is realized via numerous mechanisms, affecting both ROS producing and expression/activity of ROS eliminating enzymes [156,157,158,159,160]. Ubiquitous defense networks against oxidative stress and inflammation involve the Keap-1 (Kelch-like ECH-associated protein)/Nrf2/ARE (antioxidant response element) signaling pathway, which promotes the expression of multiple antioxidant genes containing ARE in their promoter region (for reviews, see [18,125,126]). In different cell types, the anti-oxidative effect of SCFAs depends on activation of the Nrf2 defense pathway [18,106,156,159,160,161]. In microglia and mammary epithelial cells, butyrate-induced Nrf2 activation and oxidative stress inhibition are mediated by GPR109A [106,156].

Although Nrf2 activation is regulated at multiple levels via various signaling pathways, the excessive ROS production is a well-known Nrf2 activator and trigger for transcription of Nrf2-target antioxidant genes [162]. In the BBB, Nrf2 augments oxidative stress and preserves integrity by increasing TJ and AJ protein expression under different pathological conditions [163,164,165,166,167] (Figure 2). Silencing Nrf2 in hCMEC/D3 cells abrogated the expression of claudin-5 and VE-cadherin leading to an increase in permeability of the monolayers and to the decline of TEER [167]. Pharmacological activation of Nrf2 signaling post-brain injury significantly restored the loss of TJ proteins and prevented BBB disruption [163].

Nrf2-mediated attenuation of BBB disruption in rats following subarachnoid hemorrhage was observed after administration of mitoquinone (MitoQ) [168], a mitochondria-targeted antioxidant that exerts protective effects in many diseases associated with oxidative stress [169]. Although mitochondrial ROS can inactivate Keap1, promoting nuclear translocation of Nrf2 [170], a mitochondrial ROS scavenger also inhibits Nrf2 degradation and subsequently upregulates the antioxidant genes, thus protecting barrier integrity, as was demonstrated in various barrier-forming cells [168,171,172,173]. The precise mechanisms of the MitoQ effect on Nrf2 activity are still poorly understood.

SCFAs, being HDAC inhibitors, can activate Nrf2 via alterations of histone acetylation state [174,175]. Such a mechanism of the SCFA anti-oxidative effect has been convincingly demonstrated in different cells [106,114,174]. In high glucose-treated aortic endothelial cells, SB inhibited HDAC activity and increased the occupancy of the transcription factor aryl hydrocarbon receptor and transcriptional adaptor P300 on the Nrf2 gene promoter, elevating Nrf2 mRNA/protein expression and alleviating oxidative stress and inflammation [174]. The interaction between SB-induced HDAC inhibition and Nrf2-associated anti-oxidative capacity has also been studied in bovine mammary epithelial cells subjected to H_2_O_2_-driven oxidative stress. SB promoted Nrf2 nuclear accumulation and H3K9/14 acetylation through the AMPK signaling pathway. Chromatin immunoprecipitation assays detected that SB enhanced acetylation of histones associated with anti-oxidative genes such as Nrf2, HO-1, GCLC, GCLM, SOD1 and NQO1, leading to the increase in their transcription and oxidative stress alleviation [106]. The contribution of HDAC to Nrf2 activity was also found to be important in the restoration of the BBB integrity. In a mouse model of T2DM, HDAC3 inhibition by its specific inhibitor RGFP966 upregulated miR-200a, thereby reducing the Keap1–Nrf2 interaction and promoting Nrf2 activation, which in turn significantly ameliorated the BBB permeability and TJ protein downregulation associated with T2DM [114]. These data indicate that epigenetic modification of Nrf2 and downstream genes may be a potential mechanism of SCFA action in the restoration of the BBB integrity under oxidative stress.

The involvement of both NF-κB- and Nrf2-driven pathways in the effect of propionate at physiological concentration (1 μM) was shown in the hCMEC/D3 cell line, a human BBB in vitro model [33]. Transcriptomic analysis revealed two particular clusters of pathways regulated by propionate treatment: those involved in the non-specific inflammatory response to microbial products, including NF-κB and Toll-like receptor signaling pathways and those involved in the response to oxidative stress [33]. Propionate restrained TLR4 activation by inhibiting the expression of the accessory protein CD14 mRNA following the reduction in protein abundance on the cell surface, thereby disrupting TLR4 signaling. In addition, exposure of hCMEC/D3 cells to propionate resulted in the upregulation of a number of antioxidant genes observed in transcriptomic analysis, and these changes occur downstream of the transcription factor Nrf2. Propionate induced translocation of Nrf2 into the nucleus and diminished the level of intracellular ROS in parallel with the alleviation of the LPS-evoked marked disruption in the intracellular localization of occludin, claudin-5 and ZO-1 [33]. These data indicate that the protective effect of propionate on BBB integrity is based on the involvement of anti-inflammatory and anti-oxidative defense mechanisms.

### 8.6. SCFAs/HDAC/FoxO1/Claudin-5

HDACs are known to be activators of FoxO1, a transcription factor involved in the negative regulation of the expression of claudin-5, the most important sealing component of the TJs. Loss of HDAC activity prevents the nuclear accumulation of FoxO1, resulting in suppression of FoxO1 activity and removal of transcriptional repression of claudin-5. Pharmacological inhibition of HDAC1 activity by entinostat, a class I HDAC inhibitor, rescued claudin-5 expression in the BBB [176]. Such a mechanism can mediate SCFAs-induced enhancement of claudin-5 expression in the BBB.

### 8.7. SCFAs / HDAC / PPAR γ

**Figure 3:**
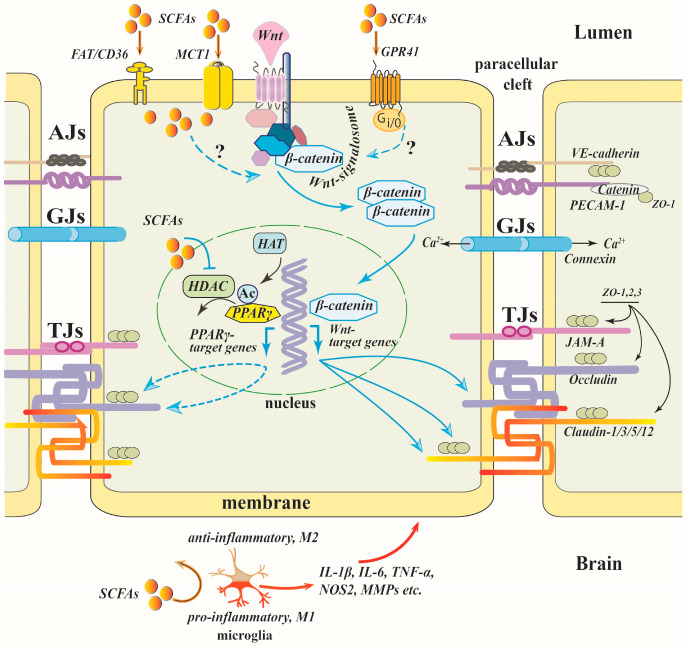
Mechanisms of SCFA effects on a brain endothelial cell (Part 2). Inhibition of HDAC by SCFAs leads to hyperacetylation of PPARγ and its translocation into the nucleus potentiating transcription of TJPs. Activation of the Wnt signaling pathway promotes translocation of β-catenin into the nucleus and upregulation of transcription of ZO-1, occludin and claudin-5. The crosstalk between SCFA signaling and the canonical Wnt/β-catenin pathway could be presumed. SCFAs can interact with microglia by binding to G-protein receptors or by inhibiting HDAC, suppressing their activation. Dotted lines—putative signaling pathways. Symbols on the scheme—see in the list of abbreviations.

Inhibition of HDAC3 may promote acetylation of peroxisome proliferator-activated receptor γ (PPARγ) thereby increasing its transcriptional activity independently of ligand [177,178] (Figure 3). In models of cerebral ischemia, both in vitro and in vivo, PPARs have been shown to contribute to the protection of the integrity of the BBB, which is partially attributed to modulating TJ protein expression [113,179,180]. In human brain microvascular endothelial cells subjected to oxygen-glucose deprivation in the presence of a specific HDAC3 inhibitor and a PPARγ antagonist, it was shown that HDAC3 inhibitor-induced reduction in increased endothelial permeability is at least partly mediated by promotion of PPARγ DNA binding activity [113]. The effect of SCFAs (a mixture of acetate, propionate and butyrate in 3:1:1 ratio) on the prevention of high-fructose diet-induced intestinal epithelial barrier impairment in mice was mimicked by a selective PPARγ agonist [181].

### 8.8. SCFAs/Myosin Light Chain Kinase

BBB “opening” depends on the endothelial actin–myosin cytoskeleton which regulates junction assembly and function. Non-muscle myosin II binds to actin, which is linked via adapter proteins to TJ proteins providing a driving power for their rearrangement. Phosphorylation of the myosin light chain (MLC) by MLCK results in the contraction of the TJPs-associated actin filaments, leading to the relocalization of the TJ proteins and intercellular barrier opening [182,183] (Figure 2). Pharmacological inhibition of MLCK prevented an increase in BBB permeability induced by pathological stimuli, as was demonstrated both in vivo [184,185] and in vitro [185]. MLCK deficiency attenuates endothelial barrier dysfunction [186]. Using primary brain microvessel endothelial cells isolated from mice null for MLCK, Beard and coauthors found that MLCK mediates IL-1β-induced claudin 5 repression and barrier dysfunction acting in a manner that promotes the nuclear translocation of β-catenin, resulting in the repression of Cldn5 gene expression [187]. Thus, myosin light chain kinase (MLCK) is now considered a crucial regulator of endothelial permeability and a potential therapeutic target in the treatment of tissue barrier dysfunction.

A link between SCFAs and MLCK was studied in the intestinal epithelium. In Caco-2 cell monolayers, SB had no effect on the expression level of several components of the TJ complex, but promoted the reassembly of TJ proteins through AMPK-dependent suppression of MLCK activity and subsequent decrease in the phosphorylation of MLC2 [188]. Data obtained on fish intestine subjected to infection showed that SB dietary supplementation maintained the intestinal epithelium integrity decreasing the level of MLCK mRNA in the intestine [161] indicating that the protective effect of SB is at least partly mediated by MLCK inhibition.

As far as we know, the relationship between SCFAs and MLCK in endothelial cells has not yet been studied. Nevertheless, given the critical importance of an MLCK-dependent mechanism in BBB function and dysregulation, the study of the potential involvement of SCFAs, mainly butyrate, in MLCK regulation in the BBB is a promising direction.

### 8.9. SCFAs/Wnt/β-Catenin

Both canonical and non-canonical Wnt pathways are involved in the regulation of BBB integrity (Figure 3). The canonical Wnt/β-catenin signaling is critically required for the formation of BBB-specific barrier properties in endothelial cells during brain angiogenesis [189] and is essential for the maintenance of the BBB integrity in the mature brain via transcriptional regulation of the expression of TJ proteins [190]. In the canonical Wnt signaling pathway, Wnt inhibits the degradation of β-catenin through its binding to Frizzled receptors, promoting its translocation into the nucleus and upregulation of genes critical for the maintenance of the BBB, such as claudin-1 and -3 [190,191]. The noncanonical β-catenin-independent Wnt pathways, which include Wnt/calcium and Wnt/planar cell polarity (PCP) pathways, contribute to the regulation of TJ complex stability and endothelial cell polarity in the BBB [192].

Despite the critical importance of the Wnt signaling pathway for the integrity of the BBB, we found only one work linking the protective effect of SCFAs in the BBB with its activation [71]. In aged mice subjected to anesthesia/surgery, SB or Lactobacillus bacteria mixture improved postoperative cognitive function, reduced BBB permeability and increased the expression of occludin, claudin-5 and ZO-1. β-Catenin protein expression in the hippocampus was significantly enhanced under both treatments, suggesting crosstalk between SCFA signaling and the canonical Wnt/β-catenin pathway [71].

## 9. Indirect Effects of SCFA on BBB Integrity

### 9.1. Systemic Inflammation

Brain endothelial cells possess mechanisms protecting them from the sensing of peripheral inflammation in order to avoid neuroinflammation (e.g., endothelial glycocalyx, low level of pro-inflammatory TNF-α, IL-6 and IL-1β cytokine receptors) [193]. However, during infectious, non-infectious and gut dysbiosis-associated systemic inflammation, a plethora of highly produced inflammatory mediators can increase BBB permeability through multiple mechanisms including activation of perivascular microglia and any NVU component (for reviews, see [5,6,37,193,194]). Excessive levels of circulating pro-inflammatory cytokines under systemic inflammation also leads to upregulation of the expression of their receptors, as well as chemokine receptors, and cellular adhesion molecules in brain endothelial cells, enforcing the sensing of inflammatory stimuli [193]. Thus, under inflammatory conditions, including age-increased inflammation, brain endothelial cells become highly vulnerable to the detrimental effects of inflammatory mediators associated with the damage of both paracellular transport and transcytosis [195].

Epidemiological data indicate that increased consumption of dietary fiber shifts microbiota composition and reduces systemic inflammation, while low-fiber diets are associated with increased inflammatory disorders [196]. SCFAs, metabolites of dietary fibers, enter the systemic circulation from the intestinal lumen and act on immune and other cell types reducing systemic inflammation. The anti-inflammatory action of SCFAs in systemic inflammatory disorders is provided by multiple mechanisms including promotion of extrathymic differentiation of regulatory T cells (Treg), inhibition of immune cell recruitment and suppression of the production of inflammatory mediators, such as TNF-α, IL-6, IL-1β, NO, eicosanoids and chemokines by immune and endothelial cells [196,197,198,199,200]. These effects can be provided by HDAC inhibition and by binding to different types of SCFA receptors which are highly expressed in immune cells. In GPR43-KO mice and in GPR43 siRNA RAW264.7 macrophages, the ameliorating effect of SB against LPS was weakened [93].

A reduction in circulating proinflammatory cytokine levels under SCFAs, mainly butyrate, was demonstrated in various models of systemic inflammation [93,201,202], indicating that SCFAs can protect the BBB indirectly by regulating immune cell activity and peripheral inflammation.

### 9.2. Microglia and Astrocytes

Many, if not all, pathological conditions of the CNS are accompanied by the activation of microglia, a key player in the development of neuroinflammation, which respond to injury by generation of numerous inflammatory mediators such as reactive free radicals, cytokines, chemokines and others (for a review, see [5]). Activated microglia, as well as pericytes and astrocytes, through inflammatory cascade amplification and oxidative stress, cause disturbances of the endothelial barrier function [203,204,205]. On the other hand, activation of microglial and other NVU cells could in turn be secondary to BBB impairment, which facilitates the penetration of multiple inflammatory factors and peripheral immune cells. In this case, microglia respond by overproduction of pro-inflammatory mediators, which leads to further destruction of the barrier [5].

Microglia appear to be highly sensitive to SCFAs. As mentioned above (Section 3), the brain effectively takes up SCFAs, and the concentration of butyrate and propionate in the brain tissue is at the same level as in the plasma. Keeping in mind that three SCFA-specific receptors, GPR43, GPR41 and GPR109A, are expressed in microglial cells [98,206], and that the involvement of HDAC in microglia activation has been demonstrated in many studies (for a review, see [207]), the SCFA–microglia interaction appears to be even more relevant for the preservation of the BBB than the direct action of SCFAs on endothelial cells (Figure 3). Interestingly, in the initial phase of the systemic inflammation microglial cells migrate from the parenchyma to the cerebral vasculature in response to chemokine CCR5 expressed by endothelial cells and produce claudin-5, making close physical contacts with endothelial cells, thereby tightening the BBB. During sustained inflammation, microglia phagocytose astrocytic endfeet leading to the impairment of the BBB [208]. The increase in vessel-associated microglia in inflammatory conditions may likely enhance the availability of plasma SCFAs to microglia.

Microglia activation is suppressed by SCFAs both in vivo and in vitro. In the mouse model of sepsis-associated encephalopathy, SB administered intragastrically for 7 days before the cecal ligation and perforation procedure protected mice against microglia activation and brain damage [156]. In primary cultured microglia, SB partially activated GPR109A, triggering the downstream Nrf2/HO-1 pathway, thereby inhibiting the oxidative stress response [156]. Other authors [67] exploring the same in vivo experimental model showed that a SCFAs mixture (acetate:propionate:butyrate, 3:1:1) attenuated microglia activation, and this effect was reversed by GLPG0974, a GPR43 antagonist. The authors suggest that the SCFA effect is mediated by the GPR43/β-arrestin-2/NF-κB signaling pathway [67].

Cerebral ischemia is a well-known trigger of microglia activation, neuroinflammation and BBB impairment. In in vitro hypoxia/ischemia conditions, such as oxygen and glucose deprivation, SB decreased proliferation of microglial cells and altered the microglia phenotype from proinflammatory M1 to anti-inflammatory M2, providing immunosuppressive and neuroprotective processes [209]. In mice subjected to MCAO, SB downregulated the expression of pro-inflammatory TNF-α and iNOS, and upregulated the expression of anti-inflammatory IL-10 in activated microglia [210]. Using the same experimental model of ischemic conditions, other authors [211] showed that post-insult treatment with SB improved neurological outcome by suppressing microglial activation and neuroinflammation. These effects of SB were mediated by inhibition of HDAC [211].

SB inhibited the LPS-stimulated secretion of pro-inflammatory IL-6 and TNF-α by primary cultured microglia [212]. Acetate, the most abundant SCFA in the blood, suppressed the activation of LPS-treated BV2 microglial cells but not when GPR43 was silenced, indicating its involvement [98]. Interestingly, if BV2 microglial cells were stimulated by amyloid-β, acetate also inhibited activation, but in contrast to LPS treatment, its effect was mediated by binding to GPR41 [213].

Alterations in the gut microbiota can impair microglia function. Germ-free rats exhibit microglia activation and increased proinflammatory cytokine levels. Propionate or butyrate treatment reduces microglial activation via HDAC1 inhibition [214]. In germ-free mice, defects in maturation, differentiation and function of microglial cells were observed. Supplementation with an SCFA mixture (acetate, propionate and butyrate) reversed microglial immaturity [19].

SCFAs also affect hypoxia-induced astrocyte activation. Both in vivo with the model of hypoxic–ischemic brain injury and in vitro on primary cultured astrocytes subjected to hypoxia, a mixture of acetate, propionate and butyrate (3:1:1) attenuated astrocyte overactivation, which was manifested as a decrease in IL-6, chemokine CCL2 and NLRP3 inflammasome expression levels [215]. It was shown that this effect is mediated by SCFA-induced overexpression of serum and glucocorticoid-induced protein kinase 1 (SGK1).

## 10. Conclusions

During long-time coevolution of mammals and their gut microbiota, close metabolic interactions between both symbiotic participants should have developed. SCFAs, end products of the gut microbiota metabolism, are used highly efficiently by the host. They not only provide intestinal homeostasis, maintaining local immunity, integrity and supporting the energy needs of colonocytes, but also exert pleiotropic beneficial effects regulating immunity, metabolism, vascular tonus, brain function and others. Many publications reviewed here show that SCFAs, along with an increase in positive neurological outcomes in different pathological conditions, recovered the impaired BBB integrity. Due to the reciprocal interaction between BBB disruption and neuropathological processes, in some cases, it is difficult to differentiate the cause and the effect. However, there is no doubt that the maintenance of the structural and functional integrity of the BBB by SCFAs is one of the main mechanisms of their neuroprotective action. SCFA-induced recovery of BBB integrity can be mediated by the direct action of SCFAs on brain endothelial cells, as evidenced by in vitro data, as well as indirectly through suppression of systemic inflammation and inhibition of microglia and astrocyte activation. Interestingly, among natural fatty acids, not only the SCFAs regulate BBB permeability. SCFAs restrict paracellular transport, while long-chain polyunsaturated fatty acids, such as docosahexaenoic and eicosapentaenoic acids, suppress transcellular permeability interfering with caveolae-mediated vesicular transport [35].

Most reviewed publications provide evidence that the mainstream mechanisms of SCFA action in the improvement of the impaired BBB is associated with the restoration of junctional complex proteins via regulation of their transcription, intercellular localization or proteolytic degradation. Although brain endothelial cells possess a strong BBB-specific molecular phenotype in comparison with peripheral vasculature [216], the effect of SCFAs on junctional complexes seems not to be specific to brain endothelium. Rather, SCFAs can be considered as ubiquitous barrier protectors in various tissues, such as intestinal, mammary, renal epithelia, peripheral and brain endothelium. Although the mechanisms of SCFA sensing and signaling are cell specific, depending on receptor/transporter expression, SCFA availability and other factors, the downstream pathways eventually result in the recovery of TJ proteins and reduction in the barrier permeability in various barrier tissues. Given the fact that oxidative stress and inflammation are the main reasons for disruption of junctional complexes and barrier leakiness, the ability of SCFAs to inhibit pro-inflammatory NF-κB and activate anti-oxidant Nrf2 pathways could probably explain their protective properties in various barrier tissues. The mechanisms of SCFA-induced improvement of the BBB integrity seem not to be based only on TJ proteins restoration. Due to anti-oxidant and anti-inflammatory effects of SCFAs, the recovery of intercellular junctions is rather a part of their overall cytoprotective effect, preventing brain endothelial cell damage and apoptosis.

Although SCFA-induced recovery of TJ proteins and BBB integrity was demonstrated in many publications, the signaling pathways mediating their effects are still poorly understood, especially the potential involvement of SCFA receptors. To date, GPR41 has been identified in brain endothelial cells, but there are no data evidencing its role in the BBB. In contrast to that, experimental data demonstrated the ability of SCFAs to modulate the expression of the BBB critical genes by inhibiting deacetylation of histones and non-histone proteins. Keeping in mind that a huge number of proteins can be acetylated at their lysine residues, new mechanisms of SCFA action in the BBB may be discovered in the future.

Administration of SCFAs, as well as the increase in their endogenous production by manipulating the gut microbiome using pro-, prebiotic and fecal transplantation, show promising therapeutic potential in the treatment of many human neurological, behavioral, and psychiatric disorders, including aging. We hope that our review, focused on both the identified and potential underlying mechanisms of SCFA action in the BBB, will help to choose prospective areas for further research on the effects of SCFAs on brain function and provide potentially therapeutic implications in the treatment of BBB hyperpermeability under different pathological conditions.

## Figures and Tables

**Table 1:** SCFA effects on the BBB in in vitro and in vivo models.

Method	SCFA	Model	Dose and Mode of SCFA Administration	Effect on BBB	Reference
In vitro	butyrate	Endothelial/glial cells co-culture	20–80 μM, 6 h incubation	↑TEER	[75]
In vitro	butyrate, propionate	hCMEC/D3 cells, LPS	1 μM, 24 h	↑TEER, ↓permeability (FITC-labeled dextran)	[33]
In vitro	propionate	hCMEC/D3 cells, LPS	1 μM, 24 h	↓NFκB, ↓TLR4 signaling pathways, ↑Nrf2-related protein expression, ↑Nrf2 translocation to the nucleus, ↑occludin, claudin-5 mRNA and protein level, ↓ROS	[33]
In vitro	butyrate, propionate	bEnd.3 cells, LPS	1 μM, 24 h	↑TEER, ↑ZO-1 and claudin-5 localization at the cell–cell junctions, ↑area occupied by mitochondria, no changes in occludin, claudin-5 and ZO-1 mRNA level	[76]
In vivo	butyrate	Stroke (MCAO)	300 mg/kg, i.p.	↓permeability (Evans blue extravasation), ↓NF-κB, ↓MMP-9, ↑claudin-5, ZO-1 protein level	[77]
In vivo	butyrate	Stroke (MCAO)	300 mg/kg, i.p.	↓permeability (serum levels of glial fibrillary acidic protein)	[29]
In vivo	butyrate	A mouse model of Parkinson’s disease	200 mg/kg, i.g.	↑occludin, ZO-1 protein level	[32]
In vivo	butyrate	Traumatic brain injury, mice	200 mg/kg, i.p.	↓permeability (Evans blue extravasation) ↑occludin, ZO-1 protein level	[30]
In vivo	acetate: propionate: butyrate 3:1:1	Sepsis (cecal ligation and puncture), mice	500 mg/kg, i.g.	↑occludin, ZO-1 protein level	[31]
In vivo	butyrate	Postoperative aged mice	i.g., (dose is not indicated)	↓permeability (FITC-labeled dextran), ↑occludin, claudin-5, ZO-1 protein level	[71]
In vivo	SCFAs mixture	High-fructose diet + chronic stress, mice	supplemented to feed, (dose is not indicated)	↓permeability (FITC-labeled dextran) ↑occludin, claudin-5, ZO-1 protein level	[7]
In vivo	monobutyrin, monovalerin	ApoE-/- rats + high-fat diet	supplemented to feed in a dose of 1%	↑occludin protein, ↑occludin, ZO-1 protein level	[78]
In vivo	butyrate	Germ-free mice	1 g/kg, i.g.	↓permeability (Evans blue extravasation, [^11^C]raclopride) ↑occludin protein level	[25]

MCAO—middle cerebral artery occlusion; i.p.—intraperitoneally; i.g.—intragastrically; TEER—transendothelial electrical resistance; hCMEC/D3—human cerebral microvascular endothelial cell line; bEnd.3 cells—murine brain endothelial cell line; ApoE—apolipoprotein E.

## Data Availability

The data underlying this article will be shared at reasonable request to the corresponding author.

## Abbreviations

**AJ**	adherens junction
**AMPK**	AMP-activated protein kinase
**ApoE**	apolipoprotein E
**ARE**	antioxidant response element
**BBB**	blood–brain barrier
**bEnd.3**	murine brain endothelial cell line
**COX2**	cyclooxygenase 2
**CNS**	central nervous system
**ERK1/2**	extracellular-signal-regulated kinase 1/2
**GCLC**	glutamate-cysteine ligase catalytic subunit
**GCLM**	glutamate-cysteine ligase regulatory subunit
**GJ**	gap junctions
**GPR**	G protein-coupled receptor
**HAT**	histone acetylase
**HDAC**	histone deacetylase
**hCMEC/D3**	human cerebral microvascular endothelial cell line
**H3K9/14**	histone
**H3**	acetylated in lysine 9/14
**HO-1**	heme oxygenase 1
**i.g.**	intragastrically
**IL-1β**	interleukin 1-β
**iNOS**	inducible NO-synthase
**i.p.**	intraperitoneally
**JAM**	junction adhesion molecule
**JNK1/2**	C-Jun N-terminal kinase
**Keap1**	Kelch-like ECH-associated protein 1
**LPS**	lipopolysaccharide
**NF-** **κB**	nuclear factor kappa B
**Nlrp3**	NOD-like receptor family pyrin domain containing 3
**NQO1**	NAD(P)H:quinone oxidoreductase-1
**Nrf2**	nuclear erythroid 2-related factor 2
**NVU**	neurovascular unit
**MCAO**	middle cerebral artery occlusion
**MCT**	monocarboxylate transporter
**MMP**	metalloproteinase
**PPARγ**	peroxisome proliferator-activated receptor gamma
**SB**	sodium butyrate
**SCFA**	short-chain fatty acid
**SOD1**	superoxide dismutase 1
**T2DM**	type 2 diabetes mellitus
**TEER**	transendothelial electrical resistance
**TJ**	tight junction
**TNFα**	tumor necrosis factor alpha
**ZO**	zonula occludens
